# Unmet need for health care services among Rohingya Refugees living in Cox’s Bazar and Bhasan Char in Bangladesh

**DOI:** 10.1371/journal.pgph.0005779

**Published:** 2026-01-21

**Authors:** Nuruzzaman Khan, Md Badsha Alam, Md Shohel Rana, Karen Block

**Affiliations:** 1 Department of Population Science, Jatiya Kabi Kazi Nazrul Islam University, Mymensingh, Bangladesh; 2 Nossal Institute for Global Health, Melbourne School of Population and Global Health, The University of Melbourne, Australia; Yale University School of Medicine, UNITED STATES OF AMERICA

## Abstract

Rohingya refugees in Bangladesh face major healthcare challenges, and access to services to those relocated to Bhasan Char is a growing global concern. This study explores the prevalence of unmet healthcare needs across physical and mental health conditions among Rohingya refugees, compare variations between camps in Cox’s Bazar and Bhasan Char, and identifies key determinants of unmet healthcare needs overall and by camp location. We analyzed data from 11,421 individuals using two surveys: the 2023 Bhasan Char Needs Assessment and the 2024 Joint Multi-Sectoral Needs Assessment (J-MSNA) in Cox’s Bazar, both conducted using similar procedures. The outcome variable was unmet healthcare needs (yes vs no), and explanatory variables included age, sex, disability level, age of household head, household size, distance to the nearest health facility from home, and mode of travel to healthcare facilities. Multivariable logistic regression models were used to explore the associations between the outcome and explanatory variables. We found approximately 10% of Rohingya refugees reported unmet healthcare needs, with the highest unmet needs observed for acute illnesses (80%), followed by preventive care (18.3%), chronic conditions (14.7%), and trauma care (4.8%). Significant variation was observed within Cox’s Bazar’s camps, with refugees in Teknaf camps having a 25% (aOR: 1.25; 95% CI: 1.07–1.46) higher odds of unmet healthcare needs compared to those in Ukhiya camps. Key determinants of unmet healthcare needs include older age, moderate to severe disabilities, and larger household size. No significant differences in unmet healthcare needs were found between residents of Cox’s Bazar and Bhasan Char. These findings highlight that, although overall differences between Bhasan Char and Cox’s Bazar were not evident, substantial inequalities exist across camps within Cox’s Bazar. This underscores the need for targeted interventions, particularly in Teknaf camps, and tailored strategies to support vulnerable groups such as those living with disabilities.

## Introduction

The mass exodus of Rohingya from Myanmar’s Rakhine State escalated in August 2017 following intense military-led violence and persecution, which the United Nations has described as an example of ethnic cleansing [1,2]. Rooted in decades of state-sponsored discrimination, statelessness under Myanmar’s 1982 Citizenship Law, and systematic exclusion from political, educational, and economic participation, the Rohingya crisis represents one of the most protracted and geopolitically complex refugee situations in South and Southeast Asia. Since 2017, Bangladesh has hosted over one million Rohingya refugees, classifying them as forcibly displaced Myanmar nationals rather than formally recognized refugees, mainly in the coastal district of Cox’s Bazar-a region already challenged by high poverty and high population density [3–6]. These camps have evolved into some of the largest and most congested refugee settlements in the world, posing enormous challenges for the provision of food, shelter, education, security, and especially healthcare. The camps are located in hilly, flood-prone terrain with inadequate sanitation networks, poor drainage, and frequent fire and landslide hazards-conditions that severely constrain public health management and humanitarian logistics. Residents are legally prohibited from formal employment or movement outside the camps, rendering them entirely dependent on assistance from the Government of Bangladesh and international humanitarian partners for their basic needs [7,8]. While the Government and agencies such as UNHCR, WHO, and IOM have provided substantial and ongoing support [5,8,9], the sustainability of this aid is increasingly threatened by competing global crises, shifting donor priorities, and a gradual decline in international political attention [10,11].

The health needs of the Rohingya population are extensive, driven by pre-existing vulnerabilities and exacerbated by the harsh conditions in the refugee camps [12–14]. Historically, they have faced significant barriers to accessing adequate healthcare services, resulting in long-term unmet medical needs due to untreated conditions [6,13,15]. This situation is compounded by physical injuries and severe psychological trauma stemming from the violence they endured in Myanmar, particularly during perilous journeys to Bangladesh [16]. Currently, approximately one million Rohingya refugees live across 33 camps in Cox’s Bazar, with densities exceeding 40,000 people per square kilometre in some areas, making these among the most crowded settlements in the world. Overcrowding, poor sanitation, inadequate nutrition, and limited access to clean water create conditions that accelerate the spread of communicable diseases and hinder effective healthcare delivery [13,17,18]. While overall demographic and facility data are collected by humanitarian agencies, consistent camp-level statistics remain limited due to security restrictions, under-reporting, and mobility constraints-factors that also impede accurate identification of unmet healthcare needs. These challenges disproportionately affect women, children, and the elderly, who are already among the most vulnerable [5,19]. Women face gender-based restrictions and caregiving burdens that limit their mobility and access to services; children are at higher biological risk of infection and malnutrition; and older adults often experience chronic illnesses and physical immobility that prevent timely care [20–22]. The prevalence of chronic conditions, such as diabetes and hypertension, is rising rapidly within this population, reflecting trends across the world [13]. Maternal and child health services remain critically underdeveloped, and mental healthcare is grossly insufficient to meet the high demand among those affected by trauma [4–6,23].

The healthcare challenges in the Rohingya refugee camps are closely tied to limited access to quality medical care, shortages of trained healthcare personnel, and inadequate infrastructure [4,6,13,24]. The doctor-to-population ratio in the camps is estimated at approximately 1 per 40,000 people-well below the World Health Organization’s recommended minimum of 1 per 1,000-and the nurse-to-population ratio is about 1 per 20,000 [25]. This critical shortage has resulted in long waiting times, overburdened staff, and limited capacity to provide continuous or specialized care. Notably, these challenges have persisted over the years, despite significant efforts by governmental and non-governmental organizations to develop a robust healthcare system, involving expenditures of millions of dollars and the establishment of healthcare facilities in each refugee camp [26]. Nevertheless, these initiatives have proven insufficient to meet the needs of a large population that grapples with low health literacy and various existing health-related challenges [27]. Moreover, over recent years, global humanitarian priorities have shifted toward newer crises-such as those in Ukraine, Gaza, and Sudan-leading to donor fatigue and funding shortfalls for protracted emergencies like the Rohingya response. These priority shifts, together with recent funding cuts by the US government, have further strained an already fragile health system, raising serious concerns about the sustainability and operational status of clinics across the camps. While specific, publicly available data on clinic closures or service suspensions remain limited, emerging reports from humanitarian actors indicate increasing service disruptions. This reduced funding has resulted in downsized operations, withdrawal of several agencies, and decreased international visibility of the Rohingya situation, thereby limiting continued health sector investments in the camps [27–29].

Adding to these challenges is the relocation of over 35,000 Rohingya refugees to Bhasan Char-an island in the Bay of Bengal located 40 kilometers from the mainland-under a Bangladesh government initiative that began in December 2020 [30–32]. This move has sparked international discussions about the implications for refugee welfare and access to healthcare services, despite government assurances that adequate facilities have been provided [30,33]. Major concerns centre on Bhasan Char’s geographic isolation, exposure to cyclones and flooding, and limited transport connectivity, which make evacuation and emergency responses difficult. Moreover, the island’s healthcare infrastructure remains rudimentary, comprising only a few small clinics with restricted referral pathways to mainland hospitals. These structural and geographic constraints raise serious concerns about the safety and sustainability of long-term resettlement, particularly for individuals with chronic illnesses, disabilities, or maternal health needs. In addition, high staff turnover, limited professional development opportunities, and restrictions on international medical recruitment further constrain service quality and continuity.

Despite this context, limited empirical research has examined how these evolving political and funding dynamics translate into health inequities within and across camps. Most existing studies focus narrowly on maternal health or service utilization among women, often overlooking broader population-level health outcomes and the determinants of unmet healthcare needs [5,6,14,15,19,20]. Furthermore, while the overall health situation in Rohingya camps has been widely reported, there remains a critical gap in understanding the extent of within-camp variation in healthcare access and the specific predictors of unmet healthcare needs, such as age, disability, household size, and proximity to facilities. Addressing this evidence gap is crucial for designing more equitable and targeted interventions.

Therefore, this study examines the prevalence of unmet healthcare needs across different physical and mental health conditions among Rohingya refugees, focusing on variations across camps in Cox’s Bazar and Bhasan Char, and identifies the key determinants of these unmet needs for both the overall population and specific camp locations.

## Methods

### Ethical considerations

Ethical approval for the original survey was granted by the relevant institutional review boards prior to data collection. Informed consent was obtained from all participants, ensuring their rights, privacy, and confidentiality. Personal identifiers were removed from the dataset before it was provided for secondary analysis, maintaining respondent anonymity. Therefore, authors had no access to information that could identify individual participants during or after data collection. However, permission to access the survey data of anonymous participants was obtained on 21 September 2024. As this study involved secondary data analysis, no further ethical approval was required. The analysis was conducted in accordance with ethical principles for research involving human subjects, in line with the Declaration of Helsinki.

### Study setting and sampling

We combined data from two surveys conducted in the Bhasan Char in 2023 and mainland Rohingya refugee camps in Cox’s Bazar conducted in 2024, encompassing both the newly established Ukhiya camps following the 2017 influx and the long-standing Teknaf camps, including Kutupalong (KRC) and Nayapara Refugee Camps (NRC). These surveys were carried out in collaboration by Research, Evaluation, and Assessment for Communities in Humanitarian Need (REACH), Agency for Technical Cooperation and Development (ACTED), Needs and Population Monitoring - International Organization for Migration (NPM-IOM), UNHCR, and other non-governmental organizations. The findings were published as part of the Bhasan Char Needs Assessment for individuals in Bhasan Char and the Joint Multi-Sectoral Needs Assessment (J-MSNA) for individuals in Cox’s Bazar. For Cox’s Bazar, the J-MSNA has been conducted since 2018, with this study focusing on data from four rounds of survey completed by 2024. In Bhasan Char, the first survey was conducted in 2022, followed by the second round conducted in 2023, which we included in our analysis. Data were collected for these surveys by REACH in partnership with ACTED. Data collectors were hired based on a gender-balanced team aiming to collect a balanced number of gender response in the Rohingya household survey.

Both surveys employed stratified random sampling techniques using the shelter mapping of the UNHCR in both UNHCR and IOM-administered camps. Data were collected by face-to-face interviews through Kobo Collect for both surveys. In Cox’s Bazar, data were collected from 33 camps, involving 3,400 households selected using a stratified random sampling method, with a 95% confidence level and a 10% margin of error at the camp level. Data were collected from the adult household representative who provided information on behalf of the household and its members, covering 18,117 individuals. In Bhasan Char, 408 households across 59 clusters were selected using a stratified random sampling approach, with a 95% confidence level and a 5% margin of error, encompassing 1,777 individuals. Detailed information about the sampling procedures has been published elsewhere [34,35].

### Analytical sample

**Fig 1:**
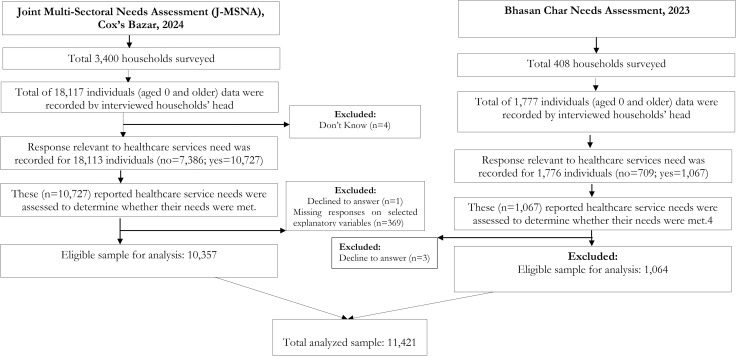
Study sample selection process to study unmet healthcare services needs among Rohingya refugees in Bangladesh, 2023-2024.

We analyzed data from a subset of 11,421 individuals from the original survey, including 10,357 from Cox’s Bazar Rohingya camps and 1064 from Bhasan Char Rohingya camps (Fig 1). Data were analyzed who met the following inclusion criteria: (i) individuals who provided information on their utilization or non-utilization of healthcare services, and (ii) individuals who provided data on their basic socio-demographic characteristics.

### Outcome variable

The outcome variable in this study was unmet healthcare need, defined as the inability to access or utilize necessary healthcare services despite a recognized need, and classified as “yes” or “no.” Relevant data were collected during the survey from the adult household representative through two questions. First, they were asked, “*During the last 3 months, did any household member have a health problem and need to access healthcare?*” with response options of “yes” or “no.” If they responded affirmatively, a follow-up question was asked: “*If yes, was he/she able to obtain healthcare when needed?*” with response options of “yes” or “no.”

Individuals were classified as having unmet healthcare needs if “yes” response recorded for them to the first question but “no” to the second. Conversely, those without unmet healthcare needs either responded “no” to the first question or “yes” to both questions.

### Explanatory variables

Several socio-demographic characteristics were included as explanatory variables, selected through a two-stage process. First, a comprehensive search was conducted to identify relevant literature for refugee settings [5,6,19,36,37]. Second, variables identified in the literature were matched with those available in the survey, and their statistical significance and multicollinearity were assessed. Variables that were both statistically significant and free of multicollinearity were included in this study. The variables considered were individuals’ age (<5 years, 5–17 years, 18–59 years, and ≥60 years), sex (male vs. female), disability level (no disabilities, moderate disabilities, and severe disabilities), age of household head (18–59 years, and ≥60 years), household size (≤4, 5–7, and ≥8), distance to the nearest health facility from home (≤10 minutes and ≥11 minutes), and mode of travel to the nearest health facility (rickshaw, bus, car, or other vehicles vs. walking). Disability level was measured using the World Health Organization’s International Classification of Functioning, Disability, and Health, covering six questions of the Washington Group Short Set (WGSS) of disability. The six questions encompassed the six functional domains such as seeing, hearing, walking, remembering, self-care, and communication-related disability [38]. There were four options for each question: (i) no difficulty, (ii) some difficulty, (iii) a lot of difficulty, and (iv) cannot do or unable to see/hear/walk/remember/self-care/communicate at all.” We reclassified the disability level for individuals as “no disabilities” if the household representative responded “no difficulty” at all to all questions, “moderate disabilities” if they answered having “some difficulty” and “severe disabilities” if they reported “a lot of difficulty” or “cannot do at all.”

Distance to the nearest health facility from participants’ home was measured based on the travel time (in minutes) to get to the nearest functional health facility by their normal mode of transportation (e.g., walking, bicycle, motorbike, bus, rickshaw, CNJ etc.).

### Statistical analysis

Descriptive statistics were used to summarize individuals’ characteristics and the distribution of unmet healthcare needs across the explanatory variables. Pearson chi-square tests assessed the significance of differences in unmet healthcare needs across these variables. Multivariable logistic regressions with robust variance were employed to explore associations between the outcome and explanatory variables. Multicollinearity was assessed prior to run each model using Variance Inflation Factor (VIF), and if any variable with VIF > 5 was excluded from the final model. Results were presented as adjusted odds ratios (aOR) with 95% confidence intervals (95% CI). The study followed the Strengthening the Reporting of Observational Studies in Epidemiology (STROBE) guidelines (S1 STROBE Checklist in S1 File), and all analyses were conducted using Stata software (version 17.0; Stata Corp, College Station, TX, USA).

## Results

### Background characteristics of the individuals

The study included 11,421 individuals, predominantly from Cox’s Bazar (n = 10,357), with the remainder from Bhasan Char (n = 1,064). In Cox’s Bazar, approximately 78% of individuals were from the Ukhiya camps (Table 1). Across the overall sample, the majority of individuals were aged 18–59 years (42.4%), with a relatively balanced gender distribution (52.6% female). Disabilities were reported by 20% of individuals. Most household heads were aged 18–59 years (84.8%), and the majority of households comprised 5–7 members (52.0%). Nearly half of the individuals (48.8%) lived within a 10-minute walking distance of a health facility, and 95.0% reported walking as their primary mode of accessing healthcare services.

**Table 1:** Background characteristics of the individuals (n = 11,421).

Characteristics	Overall, 2023–2024 (n = 11,421)		Cox’s Bazar, 2024 (n = 10,357)		Bhasan Char, 2023 (n = 1,064)	
	n	%	n	%	n	%
**Resident location in Cox’s Bazar**						
Teknaf			2245	21.7		
Ukhiya			8112	78.32		
**Individuals’ age (in years)**						
<5	2283	20.0	2008	19.4	275	25.9
5-17	3571	31.3	3261	31.5	310	29.1
18-59	4840	42.4	4398	42.5	442	41.5
60 and over	727	6.3	690	6.6	37	3.5
**Individuals’ sex**						
Male	5414	47.4	4905	47.4	509	47.9
Female	6007	52.6	5452	52.6	555	52.1
**Disability level**						
No disabilities	9239	80.9	8335	80.5	904	84.7
Moderate disabilities	1625	14.2	1489	14.4	136	12.8
Severe disabilities	557	4.9	533	5.1	24	2.5
**Age of household head (in years)**						
18-59	9680	84.8	8720	84.2	960	90.2
60 and over	1741	15.2	1637	15.8	104	9.8
**Household size**						
≤4	3029	26.5	2605	25.1	424	39.8
5-7	5933	52.0	5412	52.3	521	49.0
≥8	2459	21.5	2340	22.6	119	11.2
**Distance of nearest health facility from home**						
≤10 minutes	5569	48.8	4700	45.4	869	81.7
≥11 minutes	5852	51.2	5657	54.6	195	18.3
**Way travel to get to the nearest health facility**						
Rickshaw, bus, car and other vehicles	587	5.1	526	5.1	61	5.7
Walking	10834	94.9	9831	94.9	1003	84.3

### Prevalence of unmet healthcare service needs overall and across Camps in Cox’s Bazar

**Fig 2:**
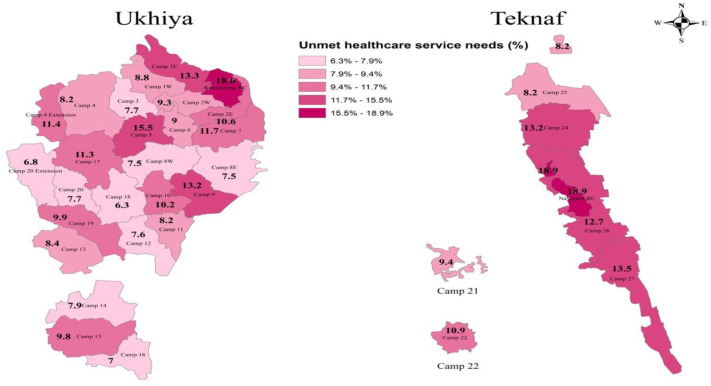
Prevalence of unmet healthcare service needs across refugee camps in Cox’s Bazar, Bangladesh (J- MSNA, 2024). Source: The map was created by the authors using ArcGIS, based on publicly available shapefiles from the Humanitarian Data Exchange (HDX, https://data.humdata.org/dataset/outline-of-camps-sites-of-rohingya-refugees-in-cox-s-bazar-bangladesh): Outline of camps and sites of Rohingya refugees in Cox’s Bazar, Bangladesh.

The overall prevalence of unmet healthcare needs among individuals was 10.3%, with Bhasan Char residents reporting a slightly lower rate of 9.9% (Table 2). However, in Cox’s Bazar, we observed significant variations in unmet healthcare service needs, with the highest prevalence recorded in Nayapara RC (18.9%) located in Teknaf camp and the lowest in Camp 18 located in Ukhiya (6.3%) (Fig 2).

**Table 2:** Prevalence of unmet healthcare service needs and types of unmet healthcare services among Rohingya refugee in Bangladesh, 2023.

Characteristics	Overall, 2023–2024		Cox’s Bazar, 2024		Bhasan Char, 2023	
	n	%	n	%	n	%
**Unmet need for healthcare services**						
No	10,246	89.7	9,287	89.7	959	90.1
Yes	1,175	10.3	1,070	10.3	105	9.9
**Types of unmet healthcare need for**						
**Preventive consultation or check-up**						
No	960	81.7	888	83.0	72	68.6
Yes	215	18.3	182	17.0	33	31.4
**Consultation or drugs for acute illness (fever, diarrhea, cough, etc.)**						
No	235	20.0	206	19.3	29	27.6
Yes	940	80.0	864	80.87	76	72.4
**Consultation or drugs for chronic illness (diabetes, hypertension, etc.)**						
No	1002	85.3	913	85.3	89	84.8
Yes	173	14.7	157	14.7	16	15.2
**Trauma care (injury, accident, conflict-related wounds)**						
No	1118	95.2	1024	95.7	95	89.5
Yes	57	4.8	46	4.3	11	10.5
**Elective, non-life saving surgery**						
No	1159	98.6	1057	98.8	102	97.1
Yes	16	1.4	13	1.2	3	2.9
**Emergency, life-saving surgery**						
No	1166	99.2	1061	99.2	105	100
Yes	9	0.8	9	0.8	0	0
**Antenatal Care or Postnatal Care services**						
No	1165	99.1	1061	99.2	104	99.0
Yes	10	0.9	9	0.8	1	1.0
**Safe delivery services**						
No	1169	99.5	1064	99.4	105	100
Yes	6	0.5	6	0.6	0	0
**Laboratory services**						
No	1162	98.9	1058	98.9	104	99.0
Yes	13	1.1	12	1.1	1	1.0
**Mental Health and Psychosocial Support (MHPSS) services**						
No	1156	98.4	1052	98.3	104	99.0
Yes	19	1.6	18	1.7	1	1.0
**Vaccination services**						
No	1169	99.5	1064	99.4	105	100
Yes	6	0.5	6	0.6	0	0
**Dental services**						
No	1168	99.4	1064	99.4	104	99.0
Yes	7	0.6	6	0.6	1	1.0

### Types of unmet healthcare services needs among Rohingya refugee in Bangladesh

Table 2 presents the types of unmet healthcare services needs among Rohingya refugee in Bangladesh. Among those with unmet needs in the overall sample, 18.3% required preventive consultations or check-ups, while 80.0% reported unmet needs for consultations or medications for acute illnesses. Additionally, 14.7% had unmet needs for consultations or medications related to chronic illnesses, such as diabetes and hypertension. Trauma care needs were unmet for 4.8%, and 1.4% reported unmet needs for elective, non-life-saving surgeries. A similar pattern was observed among Bhasan Char residents, except for preventive consultations or check-ups (31.4%), trauma care (10.5%), and elective, non-life-saving surgeries (2.9%), where unmet needs were 2–3 times higher compared to the overall and Cox’s Bazar samples.

### Distribution of unmet need for healthcare services across explanatory variables in overall and across refugees’ location of living

The distribution of unmet healthcare needs for the overall sample and across locations are presented in Table 3. In the overall sample, a higher prevalence of unmet healthcare needs was observed among individuals residing in Teknaf, older individuals, those with disabilities, households with heads of advanced age, and smaller households. This pattern was consistent in the Cox’s Bazar sample. However, in the Bhasan Char sample, higher unmet healthcare needs were reported among individuals aged 18–59 years and those from larger households.

**Table 3:** Distribution of unmet need for healthcare services across individuals’ socio-demographic characteristics, Bangladesh.

Characteristics	Overall, 2023–2024	Cox’s Bazar, 2024	Bhasan Char, 2023
**Resident location in Cox’s Bazar**		***	
Teknaf		12.5	
Ukhiya		9.7	
**Individuals’ age (in years)**	***	***	***+
<5	7.4	7.8	4.7
5-17	9.4	9.2	11.6
18-59	11.8	11.8	12.0
60 and over	13.3	13.6	8.1
**Individuals’ sex**			
Male	9.9	10.0	9.6
Female	10.6	10.7	10.1
**Disability level**	***	***	**
No disabilities	9.4	9.5	9.0
Moderate disabilities	13.0	13.0	13.2
Severe disabilities	17.1	16.7	25.0
**Age of household head (in years)**			
18-59	10.1	10.1	9.6
60 and over	11.6	11.5	12.5
**Household size**	**	***	**
≤4	10.9	11.4	7.6
5-7	10.6	10.6	10.2
≥8	8.9	8.5	16.8
**Distance of nearest health facility from home**			**
≤10 minutes	10.4	10.3	10.8
≥11 minutes	10.2	10.3	5.6
**Way travel to get to the nearest health facility**	***	***	
Rickshaw, bus, car and other vehicles	13.5	14.6	3.3
Walking	10.1	10.1	10.3

**Notes:** Row percentages are presented. *** < 0.01. ** < 0.05. P-values were obtained from Pearson chi-square test and ^+^P-value are obtained from Fisher’s Exact test.

### Factors associated with unmet healthcare services needs among Rohingya refugees in Bangladesh in overall and across their location

The multivariable logistic regression results identifying factors associated with unmet healthcare needs are presented in Table 4. No significant associations were found for unmet healthcare needs with resident location. However, individuals residing in Teknaf were 25% higher odds of reporting unmet healthcare needs compared to those in Ukhiya (aOR: 1.25, 95% CI: 1.07–1.46). In the overall sample, individuals aged 18–59 years had a 54% higher odds of reporting unmet healthcare needs (aOR: 1.54, 95% CI: 1.27–1.85), followed by those aged 60 or older (aOR: 1.43, 95% CI: 1.04–1.96) and those aged 5–17 years (aOR: 1.27, 95% CI: 1.04–1.55), compared to children under 5 years. Disability status was strongly associated with unmet healthcare needs, with individuals having moderate and severe disabilities had 27% (aOR: 1.27, 95% CI: 1.07–1.51) and 77% (aOR: 1.77, 95% CI: 1.39–2.27) increased odds, respectively, to reporting unmet needs compared to those without disabilities. Additionally, individuals who used transportation to access healthcare facilities had a 26% lower odds of unmet healthcare needs compared to those who walked (aOR: 0.74, 95% CI: 0.57–0.95). These findings were consistent across the overall Cox’s Bazar sample and its subgroups by camp (S1 Table). In the Bhasan Char sample, the results were broadly consistent, with slightly higher odds observed, except for household size, where larger households (8 or more members) had a higher odds of unmet healthcare needs. Notably, in Bhasan Char, individuals whose nearest healthcare facility was ≥ 11 minutes away reported a 52% lower odds of unmet healthcare needs compared to those living ≤10 minutes away (aOR: 0.48, 95% CI: 0.24-0.94).

**Table 4:** Results from multivariable logistic regression model with robust variance in assessing the factors associated with unmet need for healthcare services among Rohingya refugees in Bangladesh.

Characteristics	Unmet need for healthcare services					
	Overall, 2023–2024, aOR (95% CI)	P-value	Cox’s Bazar, 2024, aOR (95% CI)	P-value	Bhasan Char, 2023, aOR (95% CI)	P-value
**Resident location**						
Cox’s Bazar	1.00					
Bhasan Char	0.95 (0.77-1.19)	0.677				
**Resident location in Cox’s Bazar**						
Teknaf			1.00			
Ukhiya			1.25 (1.07-1.46)	**<0.01**		
**Individuals’ age (in years)**						
<5	1.00		1.00		1.00	
5-17	1.27 (1.04-1.55)	**0.017**	1.19 (0.97-1.46)	0.100	2.24 (1.14-4.41)	**0.019**
18-59	1.54 (1.27-1.85)	**<0.01**	1.45 (1.19-1.77)	**<0.01**	2.44 (1.25-4.73)	**<0.01**
60 and over	1.43 (1.04-1.96)	**0.029**	1.42 (1.02-1.98)	**0.038**	1.17 (0.31-4.46)	0.819
**Individuals’ sex**						
Male	1.00		1.00		1.00	
Female	1.05 (0.93-1.19)	0.432	1.05 (0.92-1.20)	0.452	1.03 (0.68-1.56)	0.887
**Disability level**						
No disabilities	1.00		1.00		1.00	
Moderate disabilities	1.27 (1.07-1.51)	**<0.01**	1.25 (1.04-1. 50)	**0.016**	1.38 (0.78-2.45)	0.268
Severe disabilities	1.77 (1.39-2.27)	**<0.01**	1.70 (1.31-2.20)	**<0.01**	2.76 (1.02-7.42)	**0.044**
**Age of household head (in years)**						
18-59	1.00		1.00		1.00	
60 and over	1.02 (0.85-1.22)	0.846	0.99 (0.82-1.20)	0.933	1.14 (0.61-2.12)	0.684
**Household size**						
≤4	1.00		**1.00**		1.00	
5-7	1.02 (0.88-1.18)	0.781	0.97 (0.84-1.13)	0.724	1.43 (0.89-2.30)	0.144
≥8	0.84 (0.69-1.01)	0.062	0.76 (0.63-0.93)	<0.01	2.58 (1.41-4.73)	**<0.01**
**Distance of nearest health facility from home**						
≤10 minutes	1.00		1.00		1.00	
≥11 minutes	0.94 (0.84-1.07)	0.364	0.98 (0.86-1.11)	0.756	0.48 (0.24-0.94)	**0.034**
**Way travel to get to the nearest health facility**						
Rickshaw, bus, car and other vehicles	1.00		1.00		1.00	
Walking	0.74 (0.57-0.95)	**0.017**	0.75 (0.57-0.98)	0.036	3.70 (0.83-16.58)	0.087

**Notes:** aOR: Adjusted Odds Ratios. 95% CI: 95% Confidence Intervals.

## Discussion

This study aimed to examine the prevalence of unmet healthcare needs among Rohingya refugees in Bangladesh residing in Cox’s Bazar and Bhasan Char, with a focus on variations across camps in Cox’s Bazar. Determinants of unmet healthcare needs were also assessed for the overall sample and across specific camp locations. No significant differences in unmet healthcare needs were found between residents of Cox’s Bazar and Bhasan Char. However, significant variations were observed across camps in Cox’s Bazar, with a 25% higher likelihood of unmet needs among residents of Teknaf camps compared to those in Ukhiya camps. Key determinants of unmet healthcare needs included older age, moderate to severe disability, larger household size, and walking is sufficient to reach the nearest healthcare facilities. These associations were observed in both the overall sample and among residents of individual locations, although the likelihoods were higher for those residing in Bhasan Char. The highest unmet need was reported for consultations and medications for acute illnesses, such as diarrhea, followed by chronic conditions like diabetes and hypertension, as well as preventive consultations or check-ups. These findings suggest that while relocation to Bhasan Char does not appear to impact healthcare access, approximately one in ten Rohingya refugees still face challenges in accessing necessary healthcare services, particularly for acute and chronic conditions. This highlights the need for targeted interventions to address these gaps.

This study found that approximately 10% of Rohingya refugees in Bangladesh reported unmet healthcare needs. While the absence of prior studies limits direct comparisons, this estimate is significantly lower than what found in global studies, which show unmet healthcare needs among refugees ranging from 26% to 88% in both low- and middle-income countries (LMICs) and high-income countries [39,40]. Several factors may explain this discrepancy. First, refugees in Bangladesh receive free healthcare services through humanitarian agencies and the Government of Bangladesh, reducing financial barriers commonly observed in host-country systems elsewhere [41]. Second, the Rohingya camps operate under a centralized humanitarian health coordination mechanism led by WHO and UNHCR, ensuring standardized care delivery across facilities [6,14,19]. Third, strong NGO involvement and proximity to primary healthcare facilities in many camps may improve access compared to urban or dispersed refugee settings [42,43]. However, this relatively lower prevalence may also reflect limited health-seeking behaviour, low expectations of care quality, and normalization of service shortages—factors that can mask deeper unmet needs [5,6,41,44]. In Bangladesh, the study identified three main areas of unmet healthcare needs: consultations and medications for acute illnesses, chronic conditions, and preventive consultations or check-ups. This finding contrasts with global evidence, where unmet needs are more commonly reported for communicable diseases and mental health issues [40]. These findings are particularly concerning given the existing high prevalence of acute conditions caused by inadequate safe water, sanitation, and hygiene (WASH) practices, as well as the rising prevalence of chronic conditions such as diabetes and hypertension [45,46]. Some of these conditions, being communicable, pose the risk of severe consequences, including rapid spread within the community. Furthermore, unmet healthcare needs for chronic conditions, especially in the context of increasing prevalence, suggest the potential for a growing burden of disease, as these conditions require consistent, long-term management. The gaps observed in Bangladesh may be attributed to differences in healthcare priorities and resource allocation. Unlike high-income settings, where mental health services and communicable disease control are often prioritized, the healthcare focus in Bangladesh appears to lean more towards addressing acute and chronic illnesses without fully addressing preventive care or long-term management of chronic diseases [47,48].

The study found no significant differences in unmet healthcare needs between refugees in Cox’s Bazar and Bhasan Char, suggesting that the recent relocation to Bhasan Char has not yet negatively affected healthcare access. However, this apparent uniformity should not be interpreted as equity in access; rather, it reflects shared structural deficiencies across both sites. These include limited-service coverage, shortages of skilled personnel, and inconsistencies in medical supply chains-systemic challenges embedded within the broader humanitarian health response, rather than issues unique to either location. This finding is particularly important within the ongoing debate about the relocation, as it underscores that the similarities between Bhasan Char and Cox’s Bazar stem from common systemic constraints rather than location-specific differences [30,33]. However, other issues, such as psychological distress and trauma, were reported to be significantly higher among Bhasan Char residents in another of our study. This may be linked to the settlement’s geographic isolation from the mainland of Bangladesh, with very limited government-maintained transportation options [44]. While the overall prevalence of unmet healthcare needs for trauma-related services was approximately 10.5%, which is about five times higher than reported for Cox’s Bazar residents, the findings indicate that, although general healthcare service availability is comparable between the two locations, there is a critical need to prioritize mental healthcare services in Bhasan Char.

Significant variations were observed across camps in Cox’s Bazar, with refugees in Teknaf having a 25% higher odds of unmet healthcare needs compared to those in Ukhiya camps. This difference is contrary to expectations, as Teknaf camps have been established for decades, with facilities built over time and there is a lower refugee density [49]. In contrast, Ukhiya camps were established after the 2017 influx of Rohingya refugees, with facility construction still ongoing and a much higher refugee density, making it the most densely populated camp in the world [1]. This discrepancy may be attributed to the greater focus by the government and non-governmental organizations on Ukhiya refugee camp’s residents, as they face more urgent healthcare needs due to the more recent traumatic events they experienced, their current living conditions, and the heightened attention from donors and humanitarian agencies towards newly relocated refugees compared to those residing in camps for a longer period [6]. However, variations in unmet healthcare needs across the camps in Ukhiya suggest that healthcare facilities are not evenly distributed within the camps, and the distance to facilities varies across different locations. Camps located farther from healthcare facilities tend to report higher unmet healthcare needs, as observed in this study and previous research on the camps [6,19].

Older refugees and those with moderate to severe disabilities faced higher odds of unmet healthcare needs, consistent with global trends where vulnerable groups experience compounded barriers to accessing care [50]. Older individuals often present with more complex health needs, including chronic illnesses, for which this study identified higher unmet demand [51]. In refugee settings, healthcare systems are often focused on addressing acute conditions and immediate care needs, with limited resources dedicated to managing chronic illnesses [52]. This lack of specialized care likely contributes to the observed trend among older refugees. Similarly, refugees with disabilities, who represent approximately 20% of the total refugee population in Bangladesh, face additional challenges. Physical inaccessibility of healthcare facilities is particularly acute in the hilly terrain of the Rohingya camps, compounding the difficulty of receiving care [53]. This is further exacerbated by stigma, a lack of specialized healthcare services, and insufficient access to assistive devices [54]. The intersection of age, disability, and refugee status magnifies these challenges, creating a cycle of unmet needs that underscores the importance of inclusive healthcare planning. Moreover, these overlapping vulnerabilities highlight the importance of inclusive, disability-sensitive, and age-responsive healthcare programming that extends beyond basic service provision.

The higher unmet healthcare needs among larger households in Bhasan Char align with global trends, potentially reflecting economic constraints that limit healthcare access for larger families [47]. However, this finding contrasts with the lower likelihood of unmet needs among larger households in Cox’s Bazar camps. This discrepancy suggests differing mechanisms influencing healthcare access between the two settings, possibly linked to variations in service availability, economic opportunities, or household-level coping strategies. Similarly, refugees in Bhasan Char living closer to healthcare facilities reported higher unmet needs, contrary to the findings in Cox’s Bazar camps. This counterintuitive result may stem from overcrowding or overutilization of nearby facilities, leading to delays, resource shortages, or inadequate service provision. Similar patterns have been observed in urban slum settings globally, where proximity does not consistently equate to better access [43,55]. These findings highlight the need for further research to unravel the underlying causes and inform targeted interventions to address these disparities effectively.

### Strengths and limitations

This study has several strengths and a few limitations. The main strength lies in its comprehensive examination of unmet healthcare needs among Rohingya refugees in Bangladesh, covering both Cox’s Bazar and Bhasan Char. Secondly, the large sample size from representative sample collected by the international recognized organizations through comprehensive procedures, inclusion of multiple camps, and use of advanced statistical analysis enhance the generalizability of the findings within the refugee population. Additionally, the study using high-quality data identifies specific areas of unmet needs, such as consultations for acute and chronic conditions, mental health care and preventive care, offering valuable insights for healthcare interventions and policy development. However, there are several mentionable limitations. First, because the study used cross-sectional data, causal relationships between predictors and unmet healthcare needs cannot be inferred. Second, the findings may not be fully generalizable beyond the two sites studied—Cox’s Bazar and Bhasan Char—since both are unique in their humanitarian governance, service coordination, and population composition. Refugee populations in other contexts, or even in future phases of the Rohingya response, may face different healthcare challenges shaped by funding levels, political priorities, or local implementation capacity. Third, we pooled data from two surveys conducted one year apart, which may have introduced minor variations, although such differences are likely negligible. Moreover, although both surveys employed stratified random sampling based on UNHCR shelter mapping and identical data collection procedures, with a 95% confidence level and a 10% margin of error at the camp level, there were differences in sample size between Cox’s Bazar and Bhasan Char. In addition, some degree of non-response or self-selection bias may have occurred, particularly if severely ill or less accessible households were underrepresented. Such biases may have led to an underestimation of the true magnitude of unmet healthcare needs and reduced the ability to detect smaller effects, especially in Bhasan Char. However, we conducted separate analyses by location to mitigate this issue. Lastly, the reliance on self-reported data introduces the potential for recall and social desirability biases, which could affect the accuracy of responses.

## Conclusion

This study highlights persistent unmet healthcare needs among Rohingya refugees in Bangladesh, with about one in ten individuals unable to access essential care. The greatest gaps were seen in services for acute illnesses, chronic diseases, and preventive care. While overall access was similar between Cox’s Bazar and Bhasan Char, notable disparities were found within Cox’s Bazar-particularly in Teknaf camps, where unmet needs were 25% higher odds than in Ukhiya. Vulnerable groups such as older adults, persons with disabilities, and larger households were most affected. These findings call for tailored interventions, including community-based outreach and mobile clinics for those with mobility challenges, integration of chronic disease management into primary care, and targeted resource allocation to high-need camps like Teknaf. Strengthening collaboration between humanitarian agencies and the Government of Bangladesh, ensuring long-term workforce development and predictable funding, and integrating refugee health services into the national system are crucial to achieving equitable and sustainable healthcare.

## Supporting information

S1 Table Results from multivariable logistic regressions with robust variance in assessing the relationship of unmet needs for healthcare services among Rohingya refugees living in Cox’s Bazar, 2024. (DOCX)

S1 File STROBE Checklist of items that should be included in reports of observational studies. (DOCX)
