# Supplementary material for: Unmet need for health care services among Rohingya Refugees living in Cox’s Bazar and Bhasan Char in Bangladesh
Source: PLOS Glob Public Health. 2026 Jan 21;6(1):e0005779. doi: 10.1371/journal.pgph.0005779 (PMC12822995; doi:10.1371/journal.pgph.0005779)
Supplement: S1 Table — (DOCX) [file pgph.0005779.s001.docx]

**S1 Table. Results from multivariable logistic regressions with robust variance in assessing the relationship of unmet needs for healthcare services among Rohingya refugees living in Cox’s Bazar, 2024**

| **Characteristics** | **Teknaf (n= 2,245)** | | **Ukhiya (n= 8,112)** | |
| --- | --- | --- | --- | --- |
|  | **aOR (95% CI)** | **P-value** | **aOR (95% CI)** | **P-value** |
| **Individuals’ age (in years)** |  |  |  |  |
| <5 | 1.00 |  | 1.00 |  |
| 5-17 | 1.17 (0.77-1.80) | 0.458 | 1.18 (0.93-1.49) | 0.173 |
| 18-59 | 1.62 (1.08-2.42) | **0.018** | 1.39 (1.11-1.74) | **<0.01** |
| 60 and over | 1.39 (0.71-2.73) | 0.338 | 1.39 (0.95-2.04) | 0.087 |
| **Individuals’ sex** |  |  |  |  |
| Male | 1.00 |  | 1.00 |  |
| Female | 1.20 (0.93-1.55) | 0.163 | 1.01 (0.87-1.17) | 0.928 |
| **Disability level** |  |  |  |  |
| No disabilities | 1.00 |  | 1.00 |  |
| Moderate disabilities | 1.05 (0.73-1.50) | 0.804 | 1.33 (1.08-1.64) | **<0.01** |
| Severe disabilities | 1.09 (0.62-1.89) | 0.772 | 1.96 (1.47-2.62) | **<0.01** |
| **Age of household head (in years)** |  |  |  |  |
| 18-59 | 1.00 |  | 1.00 |  |
| 60 and over | 1.03 (0.72-1.48) | 0.864 | 0.99 (0.79-1.24) | 0.906 |
| **Household size** |  |  |  |  |
| ≤4 | 1.00 |  | 1.00 |  |
| 5-7 | 0.91 (0.67-1.23) | 0.531 | 1.01 (0.84-1.20) | 0.930 |
| ≥8 | 0.60 (0.41-0.89) | **0.011** | 0.84 (0.67-1.05) | 0.124 |
| **Distance of nearest health facility from home** | 1.00 |  |  |  |
| ≤10 minutes | 1.00 |  | 1.00 |  |
| ≥11 minutes | 0.73 (0.57-0.94) | **0.016** | 1.07 (0.92-1.25) | 0.355 |
| **Way travel to get to the nearest health facility** |  |  |  |  |
| Rickshaw, bus, car and other vehicles | 1.00 |  | 1.00 |  |
| Walking | 0.88 (0.63-1.24) | 0.466 | 0.55 (0.36-0.84) | **<0.01** |

**Notes:** aOR: Adjusted Odds Ratios. 95% CI: 95% Confidence Intervals.
